# The participatory development of a national core set of person-centred diabetes outcome constructs for use in routine diabetes care across healthcare sectors

**DOI:** 10.1186/s40900-021-00309-7

**Published:** 2021-09-10

**Authors:** Soren Eik Skovlund, Lise H. Troelsen, Lotte Klim, Poul Erik Jakobsen, Niels Ejskjaer

**Affiliations:** 1 Department of Clinical Medicine, Aalborg University, Sønderskovvej 15, 9000 Aalborg, Denmark; 2 Department of Endocrinology, Aalborg University Hospital, Aalborg, Denmark; 3 Danish Group for European Patients’ Academy on Therapeutic Innovation (EUPATI), Copenhagen, Denmark; 4 Steno Diabetes Center North Denmark, Aalborg University Hospital, Aalborg, Denmark

**Keywords:** Health outcomes, Diabetes outcomes, User participation, Patient involvement, Participatory research, Qualitative research, Patient and public involvement (PPI)

## Abstract

**Background:**

This study sought to utilise participatory research methods to identify the perspectives of people with diabetes regarding which diabetes outcomes were most important to them. These findings were then used to support an expert working group representing multiple health sectors and healthcare disciplines and people with diabetes to establish a core set of patient-important outcome constructs for use in routine diabetes care.

**Methods:**

26 people with diabetes and family members were recruited through purposive sampling to participate in interviews, focus groups, voting and plenary activities in order to be part of identifying outcome constructs. Content and qualitative analysis methods were used with literature reviews to inform a national multi-stakeholder consensus process for a core set of person-centred diabetes outcome constructs to be used in routine diabetes care across health care settings.

**Results:**

21 people with diabetes and 5 family members representing type 1 and 2 diabetes and a range of age groups, treatment regimens and disease burden identified the following patient-reported outcome constructs as an important supplement to clinical indicators for outcome assessment in routine diabetes care: self-rated health, psychological well-being, diabetes related emotional distress and quality of life, symptom distress, treatment burden, blood sugar regulation and hypoglycemia burden, confidence in self-management and confidence in access to person-centred care and support. Consensus was reached by a national multi-stakeholder expert group to adopt measures of these constructs as a national core diabetes outcome set for use in routine value-based diabetes care.

**Conclusions:**

We found that patient-reported outcome (PRO) constructs and clinical indicators are needed in core diabetes outcome sets to evaluate outcomes of diabetes care which reflect key needs and priorities of people with diabetes. The incorporation of patient-reported outcome constructs should be considered complementary to clinical indicators in multi-stakeholder value-based health care strategies. We found participatory research methods were useful in facilitating the identification of a core prioritised set of diabetes outcome constructs for routine value-based diabetes care. The use of our method for involving patients may be useful for similar efforts in other disease areas aimed at defining suitable outcomes of person-centred value-based care. Future research should focus on developing acceptable and psychometrically valid measurement instruments to evaluate these outcome constructs as part of routine diabetes care.

**Supplementary Information:**

The online version contains supplementary material available at 10.1186/s40900-021-00309-7.

## Plain English Summary

Outcome of diabetes care is mainly measured using clinical indicators such as long-term blood sugar (A1c), cholesterol, blood pressure, occurrence of diabetes complications and need for hospitalisation. Other factors such as quality of life, well-being, treatment burden and other psychosocial issues are of high importance to people with diabetes and their family members, but these factors are often not included in outcome evaluations and there is a lack of agreement about what to measure.

This study set out to define how to measure outcomes of diabetes care that both adequately reflects priorities and needs of people with diabetes and contributes to ongoing improvement.

Twenty-one people with diabetes and 5 family members were invited to take part in interview and workshop activities to identify their views and priority outcomes. Results were summarised and used to guide the establishment of a national core set of diabetes outcomes.

In addition to clinical indicators, the following core outcomes were identified as important in order to adequately evaluate outcomes of diabetes care which matter to people with diabetes: self-reported health, psychological well-being, diabetes-related emotional distress, diabetes-related quality of life, symptom distress, treatment burden, impact of hypoglycemia, confidence in self-management and access to person-centred diabetes care and support. Involvement of people with diabetes helped fill important gaps in knowledge about how to measure outcomes of diabetes care that matter to people with diabetes. Future research is needed involving people with diabetes as partners in identifying how to best measure these outcomes as part of routine diabetes care.

**Supplementary Information:**

The online version contains supplementary material available at 10.1186/s40900-021-00309-7.

## Background

During the past decade, increasing focus has been placed on the importance of improving chronic care delivery through measurement of person-centred, patient-important health outcomes [1–5]. In several disease areas, multi-stakeholder international efforts have been undertaken to identify core outcome sets [6] or core standard measurement frameworks [7] which reflect both clinical and patient priorities. The Measurement of Outcomes Hierarchy has been applied as a new outcomes-driven measurement framework for use in implementing value-based healthcare (VBHC) and outcome driven care improvement in both the US and Europe [8]. The framework extends the traditional focus of outcome assessment from health status (tier 1) to include outcome indicators pertaining to recovery and treatment process (tier 2) as well as broader care system factors influencing sustainability of outcomes (tier 3). This expanded outcome measurement hierarchy has been suggested as a way to broaden how health care professionals (HCPs) monitor and benchmark themselves to improve effectiveness [8].

As the framework was not designed specifically for chronic illness care, two core areas have been highlighted as pivotal for the successful use of the outcomes hierarchy in conditions such as diabetes: (1) The systematic involvement of a people directly affected by the condition (i.e. patients and their family members) in the development of these outcome measures, and (2) the implementation of measurement approaches which are sensitive enough to capture each individual person’s needs and goals so they become useful tools for improving individual quality of care [9].

This study is part of the national Danish Value Based Health Care (VBHC) and Patient Reported Outcome (PRO) Diabetes Project (VBHC-PRO-DIA) undertaken by Region North Denmark under the auspices of the Danish Health Care Regions from 2017 to 2021. The project’s aims were to design and evaluate a new clinically anchored model for large-scale implementation of value-based diabetes care in Denmark in partnership with PWD and other stakeholders. The 3 main phases of the project were: (I) Identification of a core set of diabetes outcome constructs for use in value based diabetes care which could be ratified by all stakeholders to be held accountable to them, (II) Development of a national PRO measurement instrument and digital solution for coordinated integration of outcome assessment across health sectors [10–12] and (III) Assess feasibility and effectiveness and identify requirements for sustained implementation in practice [13].

This study represents the completion of phase I through the use of participatory and qualitative methods [14–17] designed to capture and apply insights of PWD comprehensively.

When this study was undertaken, no other research was identified in the literature which defined a set of diabetes outcomes which was (1) prioritized by PWD and FM based on personal importance and life priorities, (2) appropriate for use across care settings (i.e. both primary, secondary care and community care), (3) feasible and value-adding for use in individual care and (4) developed in concordance with the outcomes hierarchy of the value based framework.

## Methods

The specific objectives of this study were to

1. characterise the perspectives of PWD and FM regarding what outcome constructs are important and required for adequate ongoing evaluation of outcomes of diabetes care and
2. establish a national core set of diabetes outcome constructs for type 1 and 2 diabetes which reflects person-centred diabetes research and is ratified by PWD, HCP and payer stakeholders.

While extensive research has been done on the lived experience of PWD [18–20] and the use of PRO to measure non-clinical outcomes in diabetes [21, 22], the aim of this study was to fill specific knowledge gaps regarding PWD’s perspectives on what outcome constructs reflect their priorities, have relevance and add value for use in routine care to improve care quality.

The methodology was conducted in two steps:

- Step 1: A half-day multi-method workshop event with 21 PWD and 5 FM which included participation in written exercises, interviews, focus groups, group work activities and plenary sessions with the goal to involve PWD as equal partners in the process. The purpose of this step was to generate a comprehensive core set of the diabetes outcome constructs identified as important and required by PWD.
- Step 2: Informed by Step 1, a consensus process was undertaken with a multi-disciplinary expert working group to establish an implementation of a broadly ratified core set of outcome constructs.

Four key criteria used for development of core outcome sets in other diseases were applied: (1) Clear definition of target population and settings for use of the outcomes, (2) balanced representation of all relevant stakeholders (users and beneficiaries of outcomes) in the process, (3) systematic involvement of end-users (PWD) and literature research to identify candidate outcomes guided by empirical evidence and (4) pre-specification of judgement criteria for outcomes and procedural steps for reaching consensus [6].

### Method for user participation

We applied a systematic pre-planned stepwise method for involvement of PWD. PWD were involved in planning the participatory activities, documentation, execution and reporting in concordance with general frameworks for patient involvement in research [3]. We used key quality criteria for patient involvement under development at the time of the study including; Shared purpose and aims; Clear roles and responsibilities; Respect and diversity; Support for all to have capacity for engagement; and Representativeness [14, 15] for involvement reflecting cross-themes across multiple frameworks for patient and public involvement. We used qualitative methods for content and thematic analysis to structure insights and strengthen rigor, transparency and validity of insights derived from user participation [4, 23]. Informed consent was obtained from all involved PWD and FM.

The PWD workshop consisted of a carefully scripted set of steps each designed to facilitate the learning process and fill specific gaps regarding perspectives of PWD and FM on priority outcome constructs suitable for use in the implementation of value-based diabetes care. The workshop involved four steps with each step reflecting a core research question which mirrored a tier of the outcome measurement hierarchy [8] (see Additional file 1 for workshop agenda).

PWD and FM were recruited by phone by the study nurse.

Purposive sampling was done to ensure diverse participant representation according to factors which were identified by the research team and user participants as important to maximise validity and generalizability of results. The study nurse obtained contact details for people with diabetes eligible for the study through collaboration with the local diabetes patient organization and through direct communication with people with diabetes seen in the diabetes clinic during the course of recruitment.

The study nurse spoke with each participant on the phone to identify background, interests and potential information as well as support requirements to fully participate in and contribute to the project. 21 PWD and 5 FM took part in the user workshop. FM were recruited through invited PWD. Diversity targets regarding type of diabetes, gender, age, treatment modality (oral medication, GLP-1, insulin pen and pump), number and types of diabetes complications (neuropathy, cardiovascular, gastrointestinal, sleep symptoms) and care setting (general practice and hospital settings) were achieved (Table 1). PWD with both no, some and considerable experience with patient advocacy were included. A survey with open-ended questions (Additional file 4) was completed by participants as a pre-assignment prior to the workshop. This survey was given so that participants could reflect and prepare to address key objectives prior to the workshop and allowed researchers to collect individual qualitative data about each participant’s perspectives on the research questions before being influenced by group dynamics during the workshop event. Two persons with type 1 diabetes and two with type 2 diabetes were interviewed using the same research questions used in the survey and focus groups.

**Table 1:** Description of participants in the workshop (people with diabetes and family members)

Respondent type	Type 1 diabetes	Type 2 diabetes	Family members
Total	8	13	5 (2 T1, 3 T2)
Women/men	4/4	7/6	1/4
Age 18–60 years	7	4	0
Age > 60 years	1	9	5
Pen/pump	4/4	7/0	4/1
Tablet or no medication	0	6	2
Diabetes duration ≤ 10	3	6	1
Diabetes duration > 10 years	5	7	4
Complications: 0/1/more than 1	5/1/2	11/2/0	–
Co-morbidities	2	11	–
Primary point of care: Primary care/hospital setting	0/8	11/2	–

The 4.5-h long workshop agenda incorporated a mix of focus group, plenary and co-creation group work activities (Additional file 1). PWD and FM were split into five units for focus groups and group work according to similarities in treatment modality, type of diabetes and disease progression. The main characteristics of the 5 groups were insulin pen users (type 1), insulin pump users (type 1), non-insulin users (type 2) GLP-1 and/or insulin users (Type 2, 4) FM.

The first session introduced the aims of the national project for Value Based Health Care and Patient Reported Outcomes in Diabetes (VBHC-PRO-DIA). The aim was to “improve health and quality of life outcomes for PWD by developing a practical outcome measurement tool with PWD to ensure a greater focus on the individual needs and priorities of individual PWD.

The specific objectives of the workshop were to identify the perspectives of the participants on what diabetes outcome constructs would be most important to measure.

The second session consisted of 5 parallel moderated focus groups of one hour each in which participants shared how they perceived diabetes impacted them, how their treatment affected them and their personal goals and measures of care success. The group moderators, 2 diabetes nurses, 2 diabetes physicians and 1 PWD experienced with research advocacy and qualitative research, were trained in the use of the moderation guide defined for the purpose. The product of discussion from each focus group session were collected and summarised.

The third session was an interactive group work activity that prioritised and refined the listing of the most important diabetes outcomes and factors (care, support, environment) for achieving long-term treatment success. Results of each group’s work was collected on flipcharts and notepads and shared in plenary to identify similarities and differences in outcome topic priorities. The detailed outcomes of all 5 working groups were synthesized by the 5 group moderators into an overview which was used as a basis for a town hall discussion that involved active participation of everyone in order to achieve consensus on a comprehensive list. Participants were involved in identifying which topics and constructs were applicable to all PWD and which were applicable only to a specific subgroup of patients.

In the fourth session, PWD and FM discussed top priority outcome constructs and how these constructs could be best feasibly measured in order to improve diabetes care. Notes and transcriptions were collected from moderators, notetakers and individual participants at the end of the meeting. These were consolidated to allow for integrated analysis and summary. Group moderators and qualitative researchers met to discuss outcomes and verify group perceptions of validity, concept saturation and comprehensiveness of the summarised findings. During and at the end of the meeting participants were invited to share feedback regarding the process. Moderators of the individual workshops were two senior diabetes physicians, two diabetes specialist nurses and one experienced user expert with diabetes who had undergone formal training in user involvement and qualitative research. The overall facilitator and supervising moderator of the workshops was the first author, who has more than 15 years of experience with qualitative research within the area of diabetes psychology and outcomes research. All group moderators were trained by the first author using a detailed moderation guide and working meetings to ensure shared understanding of the methods and aims of each session and overall study.

### Use of qualitative methods to structure insights

To obtain a more nuanced and rigorous documentation of the workshop results, all free-text data from participants were analysed using a tailored stepwise coding process [24, 25]. Individual pre-assignments, interview transcripts and notes, post-its, transcripts, and flip-chart outputs from group and plenary activities were compiled and categorized. Individual and group level data were reviewed in their entirety and coded separately and then combined in an iterative process using mainly a semantic approach.

Relationships and overlaps between codes were mapped and core subthemes and constructs were identified with consistency checks across individual versus group level data using the four pre-defined research questions pertaining to impact, goals, treatment and long-term success (as listed in Table 2) as the overall structure. Coding was done by two coders until interrater agreement was confirmed. Outcome themes and constructs were checked against all codes and raw data. Illustration of quotes and basic coding is shown in Table 3. While the main set of identified constructs identified were shared for feedback and discussion during the workshop activity, a detailed summary with were shared after completion of the analysis with workshop participants for feedback and verification. A list of candidate outcome constructs was defined for use by the national working group on development of a national core set of outcomes supported by a supportive literature view pertaining to fulfillment by each construct of criteria for use as outcome.

**Table 2:** Research questions to guide identification of priority outcome constructs

1	How do PWD and FM experience diabetes mostly impacting their lives, psychologically, physically and socially?
2	How do PWD and FM describe their key goals and success criteria for their diabetes care? What are the outcomes of care they consider relevant? How do PWD suggest or believe these could be measured?
3	Do people experience negative impacts of their diabetes treatment and if so which?
4	What do PWD believe is required for their diabetes care to be successful in the long run?

**Table 3:** Illustration of coding

Outcome category	Examples of individual quotes (sample codes)	Examples of group session results/moderator notes. (sample codes)
Physical impact	“I have some problems which I think really many of us somewhat old men with diabetes have, that is sexual problems—it is in reality the thing that worries me the most—that I am most sad about in my situation.” (Symptom distress- sexual dysfunction)	“Focus on being asked about sexual function by hospital and general practitioner” (Symptom distress—sexual dysfunction) “nocturnal hypoglycemia causes poor sleep and tiredness” (symptom distress—sleep, impact of hypoglycemia)
Psychological impact	“Risk of complications affect me a lot. I worry if I will get them. I don’t think I actually will, but, in any case, I worry about it. The worry is there all the time” (diabetes stress—worry about complications)	“It’s experienced as a big part of life to always have to plan—it’s a challenge to never have a day off (treatment burden—constant demand)
Social impact	“It affects me a lot that when I am with other people that I always have to have my gear close to me. I have a pump and the bag always has to be close to me. I can’t just let it be and it affects me all the time.” (social impact—impaired enjoyment)	“Lack of understanding from others” (social impact—lack of understanding) “Feeling different. always having to explain oneself” to others (social impact—impaired enjoyment)
Desired outcomes/goals to achieve	“Feeling I got the handle on my carb intake, my insulin consumption and activity level (yesterday, today and tomorrow) without feeling that my life is controlled by numbers”	“To get a more stable blood sugar” (blood sugar regulation—stable blood sugar) “Getting better sleep” (symptom distress—sleep)
Disutility of treatment	“Frustrating to have to make finger pricks several times daily and constantly think about my blood sugar levels and the insecurity that comes with it—and that my fingers are ruined” (treatment burden—glucose measurement)	“Its difficult to always remember to have BG device, insulin, needles, and juice/food” (Treatment burden—glucose measurement/medication management) “Always having to be on—Burn-out” (Diabetes related distress, treatment burden)
Requirements for achieving good long-term care results	“The diabetes group course I went to was a tremendous success. It meant the world to me, but I know many PWD who have not attended one. You learn to regulate the three elements; Physical activity, eating and medicine.” (Access—social-motivational support (community))	“PWD want better communication in care and in general language” (Access—person-centred communication) “Better diabetes know-how in primary care” (Access—quality care) “relevant technology is not limited due to budget gaps/silo thinking” (Access—needed technology)

### Methodology for the national expert meeting on diabetes outcome constructs

The objective of the subsequent national meeting was to reach consensus on a core set of patient-important outcome constructs for use in the Danish value-based diabetes care program. Stakeholders were selected by the health authority to ensure national and appropriately balanced multi-stakeholder representation. The stakeholders were PWD, municipality diabetes education providers, clinicians (primary care physicians, diabetes nurses and physicians), public payers, authorities and researchers in health economics and health outcomes research (participants are listed in Additional file 2).

The meeting agenda involved a sequence of working sessions structured around the definition, rating and prioritisation of candidate outcome constructs that had three main sections:

- Section 1: Participants reviewed a gross list of candidate diabetes outcome constructs with the opportunity to remove, add or adjust candidate constructs prior to the prioritisation process.
- Section 2: Participants rated candidate outcome constructs on a scale from 1 to 10 individually and then in group-based consensus discussions on (1) Importance and relevance of the construct to PWD, (2) Mutability of constructs in response to a multi-level support system (systemic care perspective) which took into consideration individual, interpersonal, clinical, community factors as well as community level interventions and impact, and (3) Significance of the construct for the overall health assessment of the PWD.
- Section 3: Group and plenary discussions led to consensus on the outcome constructs to be prioritised for inclusion in the core outcome set (agenda is listed in Additional file 3).

## Results

### Results of the user participation workshop

#### Perceived impacts of diabetes

PWD reported major impacts of diabetes on social, psychological and physical health. There was significant variation in how each person felt diabetes impacted them most. PWD with more well-regulated diabetes reported no physical problems related to their diabetes while others reported major distress due to sleep problems, neuropathic pain, gastrointestinal symptoms related to treatment, fatigue, sexual problems, and symptoms of both hypo- and hyperglycemia. The physical health problems were related to late-stage diabetes complications, suboptimal glycemic control and adverse effects of medicines.

PWD reported the main negative social impact of diabetes was caused by the need for a restrictive lifestyle, constant demands for attention to self-management and frustrations resulting from a lack of understanding about diabetes from other people (e.g. one PWD expressed it as “having to always explain to people what I eat and how I eat”).

For those treated with insulin, the specific negative impacts were associated with hypoglycemia and their ability to take part in and enjoy social life.

Fear and ongoing worry about late-stage complications were the psychological impacts most frequently noted. Among insulin users, hypoglycemia, coping with the risk of hypoglycemia and other associated worries were highlighted as major negative psychological impacts. The experience of guilt and inadequacy regarding self-management were found to have important psychological impacts.

### The diabetes outcome constructs most important to PWD and FM

Table 4 summarises goals and desired outcomes identified as most important to the PWD and FM. The overarching desired goal expressed by participants was to approach normalcy in life while feeling able to manage one’s diabetes and health well. Physically, the goal was to preserve physical health and functioning by minimizing risks for disease progression measured by A1c and blood sugar stability.

**Table 4:** Outcomes of the workshop event for PWD and FM

Impact, outcomes, and factors for sustainability		
Diabetes outcome constructs considered important by PWD and FM	*Overall desired outcome*: To live as normal and healthy life as possible. Achieving a sense of normalcy and acceptance of diabetes in daily life	
	Physical	
	Prioritised outcomes	Description/underlying categories
	Physical health and well-being	Maintain physical health and functioning Minimise risk for disease progression and late stage complications
	Blood sugar regulation	A1c within range (individualised targets) Stable blood sugar/staying within range (insulin-treated)
	Diabetes symptom distress	Symptom distress related to: Neuropathic pain Sexual dysfunction Sleep problems Fatigue Cardiovascular symptoms Gastrointestinal symptoms
	Psychological	
	Mental health and well-being	Psychological well-being Mental health conditions: Depression and anxiety
	Diabetes related quality of life and emotional distress	Impact of diabetes on quality of life Worry about diabetes complications Fear of and overall burden of hypoglycemia Feeling diabetes takes up too much of daily life Frustrations due to daily self-care hassle and demands Being limited in doing activities
	Social	
	Diabetes impact on participation in and enjoyment of social activity	Limiting participation in social activities Impaired enjoyment of social activities Lack of understanding of diabetes in surroundings causing misguided attention and interference
Disutility of treatment	Burden of managing diabetes and treatment regimens	Burden of constant demand for attention to self-care Impact of lifestyle restrictions on quality of life Impact of hypoglycemia on well-being, daily life (social, work, activities), physical activity, self-care Burden of blood glucose measurement (finger pricking) Burden of medication management (hassle, injection problems, side effects)
Sustainability factors (Requirements for long-term treatment success)	Ability to manage diabetes	Confidence in ability to manage diabetes Eating healthy without feeling deprived Staying physically active Avoiding risk behaviors (i.e. smoking, alcohol) Able to navigate and use the healthcare system
	Confidence in access to quality person- centred diabetes care	*Whole person diabetes care*: Be cared for as a “whole person” with equal attention to psychosocial and biological aspects and consideration of overall health and quality of life *Person-centred interpersonal communication*: Being respected, listened to, positively encouraged, recognized for own effort and role, shared decision-making for realistic goals, person-centred language *Value-based care* Focus and tailor care around individual needs and priorities
	Access to quality of diabetes care	*Continuity of care*: Same HCP over time *Competency of HCPs*: Access to diabetes specialists *Flexibility of care*: Flexible options for care options, use of IT for flexible options for communication and sharing of own diabetes data
	Diabetes technologies which meet individual needs	Access to the technology that is needed to measure and regulate blood sugar in the best way Access to pump
	Social/motivational support for living well with diabetes in the community	Having access to social or peer support to help motivation Group based education and support activities

Minimizing symptoms which were causing distress due to existing complications was also a priority. Psychologically, achieving positive well-being as well as avoiding or dealing well with mental health problems caused by diabetes was an important outcome area. A key area of focus was the ability to have a normal daily life without interference or hassle due to diabetes tasks and avoiding a sense of exhaustion or burn-out due to the “never-ending” requirements for self-management. A key priority outcome for both types of diabetes and in all age groups related to worry and fear about getting diabetes complications. Among insulin users, hypoglycemia and the many negative impacts experienced as a result of moderate, severe and nocturnal hypoglycemia in all aspects of life were considered a top priority outcome. This group expressed the goal of achieving more stable blood sugars in order to minimize worry and impact of hypoglycemia.

### The perceived negative impacts of treatment

The key negative impacts of diabetes treatment reported by PWD overlapped with the reported psychological impacts of the disease. The main priorities to monitor for PWD were burden of self-management, symptom distress related to multiple side effects including gastro-intestinal symptoms, injection issues and hypoglycemia with the latter two issues being specific for insulin-treated PWD. Social restrictions due to lifestyle change demands were especially important to type 2 diabetes. Factors required to achieve long-term treatment success involved the ability to manage diabetes, the receiving of continuous quality whole-person-centred care, having technology tailored to individual preferences and priorities regarding areas such as lifestyle and having motivational support from the wider community if needed.

The main priority outcome constructs were agreed on by the full PWD group as relevant for both type 1 and 2 diabetes. Hypoglycemia, blood sugar fluctuations and aspects of intensive blood glucose monitoring and injection problems were specific to PWD using insulin. Importance of community level social support for lifestyle change or self-management and stigma was generally relevant but mostly expressed by those with type 2 diabetes.

The individual variation in how diabetes impacts life and the differential impacts by different treatment regimens were found to be more important than variations in impact related to type of diabetes.

### Family member perspectives on outcomes

FMs highlighted the importance of hypoglycemia, fatigue, sleep difficulties and challenges with managing multi-morbidity as proxies as well as the importance of having help for navigating the diabetes care system. A crucial way FM felt diabetes impacted them negatively related to feelings of frustration due to the PWD they lived with not allowing them to take part in their diabetes management. FMs highlighted the importance of diabetes care not only focusing on glycemic control but also actively managing sexual, gastrointestinal, cardiovascular, pain, sleep and potentially other complication-related symptoms. FMs also highlighted the importance of being able to talk openly with their family member or other peers about what each person could do to manage diabetes better. It was found that FMs would like community-level FM-friendly diabetes activities and relevant information resources such as on diabetes cooking tips.

### Ways to measure and use outcomes assessment in practice

PWD were supportive of the aim to develop a digital tool to collect PRO data as part of routine care to help focus diabetes care more effectively around each individual’s priorities and particular needs. PWD and FM identified potential additional approaches to evaluating outcomes of care:

- Use of self-recorded diary data collected via apps for dynamic outcome monitoring. E.g. number of times being frustrated with diabetes during the day, hypoglycemic episodes, specific dietary or physical habits, registration of blood sugar values, sleep and other aspects. Use of IT so health care professionals (HCP) would have easy access to individual outcomes.
- Follow-up on individual treatment goals defined and set jointly by PWD and HCP.
- Group workshops for PWD to exchange and evaluate experiences of care and share with HCPs.
- Documenting statistics for access to and use of technologies and provision of quality care.
- Consideration of involvement of FMs in evaluation activities where relevant.

Evaluative feedback from participants was positive and the opportunity to share and discuss with PWD regarding different experiences and perspectives was highlighted as a vital benefit and shared learning experience. Participants highlighted the importance of outcome measurement strategies taking into account that every PWD is affected by diabetes in a unique way and that individual experiences, resources, treatment challenges and support needs change dynamically over time.

### Consensus core set of diabetes outcome constructs.

The clinical and PRO-based outcome constructs agreed on for inclusion in the core diabetes outcome set for value-based diabetes care for PWD are listed in Table 5. This table also shows participants rating of each of the pre-defined selection criteria. The constructs measured by PRO were self-reported health, psychological well-being, diabetes related distress and quality of life, somatic symptom distress, treatment burden, hypoglycemia burden, self-management confidence, experience of quality of care and support in outcomes assessments. The clinical outcomes largely reflect the existing core outcomes integrated in the Danish clinical diabetes outcome registry at the time. Operational indicators for these clinical outcomes were already defined except for blood glucose monitoring (BGM) and continuous blood sugar measurement (CGM) data. The group asserted that further work was required to define how to use these data as supplementary outcome indicators.

**Table 5:** Patient important outcome constructs for value-based diabetes care

Clinical outcome constructs/indicators				
A1c, BGM/FMM measurements, clinician defined and registered indicators for presence of diabetes foot complications, incl. amputation, retinopathy, neuropathy, nephropathy, cardiovascular disease, ketoacidosis, severe hypoglycemia, hospitalization rate				
Patient reported outcome constructs				
Core diabetes outcome constructs	Relevance for PWD	Mutability	Clinical significance	Construct characteristics referred to in working group
*Health outcomes*				
1. Self-reported health and functioning	10	6	10	Self-reported physical health and functioning
2. Psychological well-being	10	6	10	Positive psychological well-being, mental health and risk of depression
3. Diabetes related distress	10	3	9	Diabetes-specific emotional impacts, frustrations, worries, fears, limitations, and daily burden
4. Impact of diabetes on life quality	NR	NR	NR	Impact of diabetes on quality of life beyond emotional distress. Detailing of this was out of scope
5. Somatic symptom distress	10	5	NR	Neuropathic pain. Sexual dysfunction. Sleep problems, fatigue, cognitive deficiency, Chest pain, cardiovascular symptoms, Hypo- and hyperglycemia
*Process of care and treatment*				
6. Hypoglycemic episodes requiring assistance	10	10	10	Hypoglycemic episodes requiring assistance from others were important to register through self-report to complement clinical registration
7. Burden of daily diabetes treatment	10	8	10	Perceived burden, hassles and side effects related to prescribed medical treatment regimen and requirements for daily monitoring and planning
8. Burden of hypoglycemia	9	9	9	Quality of life, emotional and behavioral burden of risks and symptoms of hypo-glycemia. Impacts on well-being, lifestyle, self-care, daily life, work/study, social and leisure life. A priority subconstruct of 7. Mostly relevant to insulin users
*Sustainability factors*				
9. Confidence in ability to perform diabetes self-management	10	7	10	Confidence in or ability to manage diabetes well: Diet, exercise, medicine, well-being, blood sugar monitoring, active role in own care decisions, navigating the care system, health competency
10. Confidence and comfort in adequate access to person-centred diabetes care	10	7	10	Feeling secure and confident in having available access to quality medical care, relevant technologies and self-management support Getting quality person-centred diabetes care; HCPs listening and communicating effectively, involving PWD in care decisions

Range for ratings of relevance for PWD, mutability and clinical significance were 1–10

*NR* not rated

It was not part of the scope of this meeting to detail or finalise operational indicators, measurement tools, data collection methods and implementation for each outcome construct. No PRO data were part of the national diabetes quality registries at the time of the meeting, so the next phase involved the design of the PRO data collection tool and the organization of its use. As an exception, the WHO-5 well-being index was approved by the working group as a suitable indicator for psychological well-being and risk of depression for both adults with type 1 and 2 diabetes based on its brevity, known measurement qualities and usage in Denmark in connection with multiple diseases.

## Discussion

The consensus that multiple PRO constructs should be part of core outcome evaluation for diabetes care marks the initiation of a national effort to implement appropriate PRO measurement tools to capture these outcomes in a way that can help improve care outcomes.

Our experience was that our systematic approach to patient involvement encompassing full cycle involvement, purposive sampling, multi-method engagement activities, qualitative analysis, literature review and use of quality criteria [14] generated valid and effective consideration of the perspective of PWD by the national working group. During evaluation at the end of the workshop, PWD expressed satisfaction with the process and noted a key benefit from participating was sharing thoughts and perspectives with other PWD and gaining personal insights related to their diabetes. The broad representation achieved from detailed purposive sampling was crucial to achieving saturation and comprehensiveness of a core set of constructs. The multi-method approach using reflection questions, focus groups, group work and plenary sessions and learning objectives helped PWD form nuanced individual and collective viewpoints on the importance and utility of different diabetes outcome constructs.

Empirical research has been found in the literature for use of the PRO constructs in health psychological [26] and clinical trials [7, 21, 27–33], outcome monitoring [32, 34], value-based care [7, 35–37] and overall relevance to person-centred diabetes management [1, 19, 21, 26, 29, 32, 38–51]. Research has also been found to support the clinical relevance of the PRO constructs of self-rated health [52], psychological well-being [53], depression [54], diabetes related distress [55, 56], diabetes impact on quality of life [57, 58], somatic symptom distress [47, 59, 60] (neuropathic pain [41], cardiovascular symptoms [41], sexual symptoms [61], sleep problems [62], foot problems [63]), burden of treatment [64–67], burden of hypoglycemia [68–70], hypoglycemia requiring assistance [71], confidence in diabetes self-management [31, 72, 73] and confidence in and access to person-centred diabetes care and support [19, 42, 74–76]. Moreover, we found supportive evidence regarding the importance of psychological well-being, confidence in self-management, treatment burden, hypoglycemia, somatic distress, and support beyond medical care to PWD [1, 7, 22, 39, 42, 43, 57, 77–86]. We did not find published studies which specifically defined a core set of patient-prioritised PRO constructs for use for outcomes evaluation as part of routine value based diabetes care. The identified PRO constructs are shown in a condensed list in Table 6 with indications of rationale and empirical support for their relevance.

**Table 6:** Condensed list of PRO diabetes outcome constructs

Abbreviated list of PRO constructs	Additional rationale
Self-reported health	Staying healthy and able to function well is a key goal Predicts care needs, outcomes and complements clinical data [52, 116]
Psychological well-being	Psychological well-being and mental health is a key goal Predicts care needs, outcomes, quality of life, risk of depression [117]
Impact on general quality of life	Minimising diabetes impact on quality of life, work/study, family/ social life, leisure life, financial situation is a key goal [19, 22, 58, 118]
Diabetes related emotional distress	Minimising diabetes distress is a key goal. Diabetes-related emotional distress (fears, worries, frustration, self-blame, exhaustion, stress due to diabetes) predicts self-care and prognosis [119, 120]
Symptom distress	Minimising symptom distress (incl. sleep [121–123], heart, pain [124–126], sexual function [61], gastrointestinal [127], feet, eyes) is a key goal. Symptoms predicts care needs, outcomes, and quality of life [47, 128]
Burden of daily managing own diabetes treatment	Minimising burden of diabetes treatment related to side-effects, medication taking, self-monitoring, requirements for planning and restrictive lifestyle is a key goal. Burden of treatment is predictive of self-management, quality of life and long-term outcomes [66, 129–132]
Burden of hypoglycemia	Minimising the burden of hypoglycemia is a key goal. Hypoglycemia impacts emotional health, self-management, daily functioning and life quality. Hypoglycemia burden predicts care needs, clinical and quality of life outcomes [69, 70, 99]
Confidence in ability to manage diabetes	Confidence in being able to self-manage diabetes is a key goal Self-efficacy predicts support needs, self-management, clinical and quality of life outcomes and complements clinical and behavioral data [133, 134]
Confidence in access to person-centred diabetes care and support	Feeling secure and confident in having quality medical care, relevant technologies and self-management support available is a key goal [42, 135]

The qualitative analysis was informed by a biopsychosocial, experiential approach with acknowledgement of the reality and importance of the individual’s subjective experience of health, illness and treatment [87].

We used the outcomes hierarchy model [8] as a conceptual framework; however, our approach to defining individual constructs was informed by multiple theoretical frameworks supported by previous empirical diabetes research. Outcome constructs of self-reported health, quality of life, symptoms, treatment impact and satisfaction are well described in the field of PRO diabetes research [21]. In analysing insights from the workshop, a wider array of empirical research pertaining to health-related constructs such as self-efficacy [72], flourishing [88, 89], illness beliefs [90], capacity [91], and a multitude of factors such as diabetes behaviors, barriers, resources, opportunities and attitudes [31] were taken into account. The outcome construct of confidence in self-management was supported by the empirical diabetes research founded on social cognitive theory [92]. The importance of systemic factors beyond healthcare was analysed in context of social-ecological theory [93] as applied to diabetes [19, 94] and other research [42]. Constructs of psychological impacts, including diabetes stress and behavioral goals, barriers and facilitators were informed by the fields of diabetes psychology and behavioral diabetes science [28, 79, 95–97].

While a wide range of health, behavioral, and psychosocial constructs have long been identified as important in diabetes [98], our study contributes by specifically eliciting both PWD, HCP and researcher’s perspectives on what constitutes an adequate core set of PRO constructs in Denmark.

While we found no other Danish initiative or research identifying a comprehensive core set of PRO constructs for routine diabetes care, we did find parallel research supporting the feasibility and utility of each of the identified PRO constructs in relation to feasibility of assessment of population prevalence and impact [19, 54, 99], predictive validity (clinical, health economic or quality of life outcomes [94, 100], theory-based definition and psychometric measurement model [29] and clinical utility [21, 101].

In the DAWN2 study, co-designed by the author, an extensive multi-stakeholder participatory process led to an internationally agreed set of indicators for person-centred diabetes care for cross-national comparison [19, 55]. Core constructs and indicators were: self-rated health (global item of EQ-5D [102]), generic quality of life (WHO-QOL-BRIEF global item [103]), psychological well-being (WHO-5 [104]), diabetes distress (Problem Areas in Diabetes (PAID-5 [105])), impact of diabetes on quality of life (Diabetes Impact of Diabetes Profile (DIDP [19, 58])), empowerment (Diabetes Empowerment Scale DAWN Short Form (DES-DSF [55]), self-management (Summary of Diabetes Self-Care activities (DSCA-6) [19, 106]), support for diabetes from medical and non-medical sources (DAWN Support for Diabetes Self-management Profile (DSDSP) [19]), access to autonomy-supportive diabetes care (Health Care Climate Questionnaire Short Form (HCCQ-SF) [19, 107]) and integrated chronic care (Patient Assessment of Chronic Illness Care–DAWN short form (PACIC-DSF)) [19, 108], use of community and education resources, and discrimination (single item) [19]. Population-based diabetes surveys in 17 countries corroborated the universal relevance of these constructs to PWD [94]. Our identified PRO constructs overlap considerably but omits use of communication and education resources and experience of discrimination. Despite our adoption of a systemic model to allow outcome constructs that could be modifiable by community services, peer support, education, medical care or technology and potentially facilitate a shared responsibility model [109], outcomes pertaining to discrimination, stigmatization or societal participation were not included in our set as it was in DAWN2.

Our finding that an overall goal for PWD is normalcy in daily life while feeling able to manage diabetes and feeling secure in having needed support from others is found by other research [110].

The involvement of the author in a concurrent development process for a global diabetes outcome standard of the International Consortium for Health Outcomes Measurement (IHCOM) [7] allowed for corroboration of the multi-national relevance of our identified core constructs. Due ﻿to pragmatic considerations, however, the ICHOM set identified WHO-5 [], PHQ-9 [111] and PAID [112] as the 3 key PROMs for diabetes outcome evaluation which cover some but not all of the constructs we identified [7]. For example, the ICHOM set does not include the outcome constructs of confidence in self-management, blood sugar stability and symptom distress.

Our outcome set differed from ICHOMs as we found strong rationale for incorporating patient reported assessment of somatic symptom distress which is not included in the ICHOM set. We found PWD considered somatic symptom distress pertaining to sexual dysfunction, pain, sleep disturbances, gastrointestinal problems and cardiovascular symptoms important to evaluate because of its significant impact on quality of life and daily functioning and self-care. We found indications that PWD would like HCPs to put greater attention to troublesome﻿ somatic distress.

A measurement of somatic symptom distress for outcome evaluation was therefore warranted from both a quality of life and medical management perspective. It was acknowledged also by HCPs that while somatic symptom distress related to complications may be less easy for a clinician to affect than glycemic control, it remains clinically relevant to pay attention to symptom distress for preventative, palliative, therapeutic and self-management support purposes.

PWD recognized that needs and priorities change dynamically over time and a comprehensive evaluation can thus play an important role by facilitating early detection, giving an overview of issues promoting health strategies and supporting consistency in quality care regardless of disease progression. The comprehensiveness of topic coverage was therefore important. The increasing access to digital health technology allows for use of computerised adaptative measurements which makes comprehensive topic coverage feasible. This can further enable the seamless differentiated use of constructs according to relevance throughout the lifespan of PWD.

A significant finding was that confidence in access to quality person-centred diabetes care that is accessible, continuous, coordinated, empathetic and whole-person centred was considered highly important by PWD. This is in line with other research which has confirmed the importance of person-centred care for psychosocial and diabetes outcomes [74, 94, 113]. Measures of perceived person- and relation-centred care and support are often not considered a part of outcomes assessment and not explicitly listed in the outcomes hierarchy framework which was originally designed for use with acute conditions. Our findings highlight that the experience of support and confidence in the accessibility of a caring health care system cannot be easily disentangled from how people adapt and perceive the way diabetes impacts them [42, 56, 114, 115]. As diabetes is a lifelong condition the quality of interpersonal relations and support has significant importance for the individual.

It was beyond the scope of this study to define the detailed constructs and components most suitable for measurement of this topic; nonetheless it was found that the experience of quality of interpersonal care and related factors is of high priority in the evaluation of diabetes care.

Further research is necessary to define the core constructs further and identify suitable patient-reported questionnaires that could be appropriate and reliable for integration in routine monitoring of diabetes outcomes and person-centred care quality.

## Limitations

Due to resource constraints, participants for this study were mainly recruited from the Aalborg area of the Region of North Denmark and it had not been possible to selectively recruit to ensure representation of different ethnic minorities. Representatives of the national patient association and PWD with years of advocacy experience were involved to facilitate broad representation of issues, however it is possible that a more balanced geographical and ethnic sample groupswould have provided a different result.

Several of the workshop moderators were HCP staff at the diabetes outpatient clinic which may have influenced the frankness of input from some PWD related to social desirability.

In this study, we focused on strengthening the voice of PWD in a structured and evidence guided way since experts were already well represented in the decision-making process. We believe that future research would benefit from more explicitly clarifying and solidifying all the different stakeholder perspectives on relevance and importance of each outcome construct.

## Conclusions

We found that in order to evaluate outcomes of diabetes care in Denmark in a way that adequately reflects the needs and priorities of PWD both clinical as well as patient-reported outcomes are needed. The core set of patient-reported outcome constructs required for inclusion in outcome evaluation were self-reported health, psychological well-being, diabetes related emotional distress and impacts of diabetes on quality of life, symptom distress, treatment burden, hypoglycemia burden, confidence in ability to manage diabetes in daily life, access to person-centred care and technology and relevant support from outside the healthcare system.

We found that the use of systematic, pre-planned stepwise methods for engagement of PWD and FM and evidence-guided structuring of patient perspectives was feasible and resulted in effective use of PWD insights which influenced the outcome of the study positively.

The next step towards implementation will be to work in continued multi-stakeholder partnership with PWD and FM to design a feasible, acceptable, valid, reliable and value-adding method to measure these patient-reported outcome constructs as an integral part of routine diabetes care and ongoing quality monitoring and improvement efforts.

## Supplementary Information

**Additional file 1**. Agenda for workshop with PWD and FM.

**Additional file 2**. Participants in national working group meeting on diabetes outcomes constructs.

**Additional file 3**. The agenda for the national expert working group meeting on value based diabetes care and development of a core diabetes outcome construct set.

**Additional file 4**. Survey completed by PWD and FM prior to workshop.

## Data Availability

The datasets used and analysed during the current study are available from the corresponding author on reasonable request.

## Abbreviations

PRO: Patient Reported Outcome
PWD: People with Diabetes
HCP: Health Care Professionals
FM: Family members of PWD
VBHC: Value-Based Health Care
VBHC-PRO-DIA: The Danish Value Based Health Care and Patient Reported Outcomes in Diabetes Programme
