# Supplementary material for: The participatory development of a national core set of person-centred diabetes outcome constructs for use in routine diabetes care across healthcare sectors
Source: Res Involv Engagem. 2021 Sep 10;7:62. doi: 10.1186/s40900-021-00309-7 (PMC8434700; doi:10.1186/s40900-021-00309-7)
Supplement: Supplementary file 1 — Additional file 1. Agenda for workshop with PWD and FM. [file 40900_2021_309_MOESM1_ESM.pdf]

Agenda for workshop with PWD and FM

3.30 pm-4.00 pm 1. Plenary Session: Aim, purpose and methods

4.00 pm- 5.00 pm 2. Focus Groups: Experiences of 1) Impact of diabetes, 2) Impact of care/therapy, 3) Personal goals/ desired outcomes of diabetes care and preconditions for success

5.00 pm-5.45 pm 3. Group Work A. Prioritising and Characterizing Key Diabetes Outcome Constructs

5.45 pm-6.35 pm 4. Group Sharing: Group presentation and sharing

6.35 pm-- 6.50 pm 5. Plenary Discussion: Towards consensus summary and shared outcome priorities

6.50 pm--7.20 pm 6. Group Work B: Co-designing ways to measure outcomes in practice to improve care

7.20 pm - 8.00 pm 7. Conclusions and next steps
