# Supplementary material for: The participatory development of a national core set of person-centred diabetes outcome constructs for use in routine diabetes care across healthcare sectors
Source: Res Involv Engagem. 2021 Sep 10;7:62. doi: 10.1186/s40900-021-00309-7 (PMC8434700; doi:10.1186/s40900-021-00309-7)
Supplement: Supplementary file 2 — Additional file 2. Participants in national working group meeting on diabetes outcomes constructs. [file 40900_2021_309_MOESM2_ESM.pdf]

Additional file 2 – Participants in national working group meeting

Advocacy and user perspective	Representative from the National Office of the Danish Diabetes Association (representing a national membership of PWD and FM in Denmark)<br>Experienced user representative with Type 1 diabetes, co-designer of study<br>Experienced user representative with Type 2 diabetes, co-designer of study
Senior diabetes physicians	North Denmark: 5 senior diabetes specialist physicians; 1 did not attend in-person<br>South Denmark: 2 senior diabetes specialist physicians;1 did not attend in-person<br>Central Denmark: 1 senior diabetes specialist physician<br>Capital Region: 1 senior diabetes specialist physician, head; Did not attending in-person
Senior diabetes nurses	North Denmark: 1 section head diabetes nurse and 1 senior diabetes specialist nurse<br>Central Denmark: 1 senior specialist diabetes nurse, did not attend in-person.
Health care payer	North Denmark Health Region: 1 Senior head, VBHC diabetes program sponsor, 1 Data and Health Economist regional project manager,
National health authorities	1 representative of the PRO secretariat of the National Health Data Board authorities

Additional file 2 – Participants in national working group meeting

Municipality care	Municipality, Northern Denmark: 1 specialist manager, municipality health promotion and rehabilitation services
Primary care physician	Primary care practice: Senior general practitioner and primary care research coordinator
Outcome researchers	1 senior PRO diabetes research scientist<br>1 health economist researcher

Participants in national working group meeting on diabetes outcomes constructs
