# Supplementary material for: The participatory development of a national core set of person-centred diabetes outcome constructs for use in routine diabetes care across healthcare sectors
Source: Res Involv Engagem. 2021 Sep 10;7:62. doi: 10.1186/s40900-021-00309-7 (PMC8434700; doi:10.1186/s40900-021-00309-7)
Supplement: Supplementary file 3 — Additional file 3. The agenda for the national expert working group meeting on value based diabetes care and development of a core diabetes outcome construct set. [file 40900_2021_309_MOESM3_ESM.pdf]

Additional file 3 – Agenda for national working group meeting

Agenda of national working group meeting for diabetes outcome constructs

9.30-10.00 Presentation by leadership of the regional healthcare system and project leads

10.00-10.20 Scientific considerations for use of patient-reported outcomes in diabetes care

10.20-10.45 Key insights from the participatory workshop with PWD and FM

10.45-11.00 Clinical outcome constructs and indicators in diabetes in Denmark

11.00-11.30 Definition of target patient population, scope and method for use of future outcomes

11.30-14.45 Ratings, evaluations, prioritization and consensus regarding a set of outcome constructs.

14.45-15.15 When and how to apply the outcome constructs in practice in Denmark?

15.15-15.30 Next steps.

The agenda for the national expert working group meeting on value based diabetes care and development of a core diabetes outcome construct set.
