# Supplementary material for: The participatory development of a national core set of person-centred diabetes outcome constructs for use in routine diabetes care across healthcare sectors
Source: Res Involv Engagem. 2021 Sep 10;7:62. doi: 10.1186/s40900-021-00309-7 (PMC8434700; doi:10.1186/s40900-021-00309-7)
Supplement: Supplementary file 4 — Additional file 4. Survey completed by PWD and FM prior to workshop. [file 40900_2021_309_MOESM4_ESM.pdf]

Additional file 4 – Survey completed by PWD and FM prior to workshop

Survey completed by PWD and FM prior to workshop.

<u>Pre-assignment for all participants</u>

Please insert text to complete the incomplete sentences below. There are no right or wrong answers.

1. The most significant way my diabetes affects my physical health is

………

…………………………………………………

and……

……………………………………………………

2. The most significant way my diabetes affects my social and psychological well-being is

………

…………………………………………………

………

…………………………………………………

and my greatest concern in relation to this is

…………………………………………………………………………………………….

3. The most important thing for me to achieve in relation to my diabetes overall is …

………

…………………………………………………

and……

……………………………………………………

4. I know that my diabetes treatment is not working well for me when

………

…………………………….

……………………………………………………………………………………….……………

…………………………………………………

And when I

feel…………………………………………………………………………………………………

………………..

5. In order for me to feel that my diabetes treatment is successful, it should

……………………………………………………………………………………….……………

……………………………………………………

and

…………………………………….………………………………………………………………

…………………………………………

(English translation is only for illustrative purposes and has not been cross-culturally adapted)
